# Associations of Whole Grain and Refined Grain Consumption With Metabolic Syndrome. A Meta-Analysis of Observational Studies

**DOI:** 10.3389/fnut.2021.695620

**Published:** 2021-07-01

**Authors:** Hongbin Guo, Jun Ding, Jieyu Liang, Yi Zhang

**Affiliations:** ^1^Department of Orthopaedics, Xiangya Hospital, Central South University, Changsha, China; ^2^Changsha Social Work College, Changsha, China

**Keywords:** whole grain, refined grain, metabolic syndrome, meta-analysis, observational study

## Abstract

**Background:** The associations of whole grain and refined grain consumption with metabolic syndrome (MetS) has been evaluated in several epidemiological studies with conflicting results. This meta-analysis was therefore employed to further investigate the above associations.

**Method:** We searched the PubMed, Web of Science and Embase database until March 2021 (without restriction for inclusion time), for observational studies on the associations of whole grain and refined grain consumption with MetS. The pooled relative risk (RR) of MetS for the highest vs. lowest category of whole grain and refined grain consumption, as well as their corresponding 95% confidence interval (CI) were calculated.

**Results:** A total of 14 observational studies, which involved seven cross-sectional and seven prospective cohort studies, were identified. Specifically, nine studies were related to whole grain consumption, and the overall multi-variable adjusted RR demonstrated that the whole grain consumption was inversely associated with MetS (RR = 0.80, 95%CI: 0.67–0.97; *P* = 0.021). With regard to refined grain consumption, 13 studies were included. The overall multi-variable adjusted RR indicated that refined grain consumption was positively associated with MetS (RR = 1.37, 95%CI: 1.02–1.84; *P* = 0.036).

**Conclusions:** The existing evidence suggests that whole grain consumption is negatively associated with MetS, whereas refined grain consumption is positively associated with MetS. Our result might be helpful to better consider the diet effect on MetS. However, more well-designed prospective cohort studies are needed to elaborate the concerned issues further.

## Introduction

The metabolic syndrome is a complex of interrelated risk factors for cardiovascular disease (CVD), diabetes and all-cause mortality. These factors include dysglycemia, raised blood pressure, elevated triglyceride levels, low high-density lipoprotein cholesterol levels, and obesity (particularly central adiposity) (1). Affecting about 25% of the population in developed countries in parallel with obesity and diabetes, MetS has been known as an important public health issue in the 21st century (2). Although the etiology of MetS is still not well-understood yet, dietary factors are considered to be associated with MetS (3–5).

Grain, a small, hard and dry seed, is composed of the endosperm, germ, and bran (6). Grain is served as the primary source of carbohydrates and global staples of diets (7). Whole grain contains more abundant and diverse nutrients with potential health benefits (fiber, vitamins, and minerals) than refined grains (8). Indeed, a potential different biological effect of whole grain and refined grain on health issues has been reported, e.g., gastric cancer (6), chronic kidney disease (9) or all-cause mortality (10), etc. Moreover, whole grain consumption was indicated to be associated with a lower risk of hypertension (11) and diabetes (12, 13), whereas refined grain consumption was associated with higher risk of diabetes (13). Moreover, some randomized control trials demonstrated that whole grain (replacing refined grain) within a weight-loss diet could reduce blood glucose (14). A whole grain-based diet could also lower the postprandial plasma insulin and triglyceride level in MetS (15). With regard to the fundamental research, the pre-germinated brown rice extract was also proved to ameliorate MetS model (16, 17).

As far as we know, the associations of whole grain and refined grain consumption with MetS has been investigated by numerous observational studies (18–31). However, their results are still controversial. The present meta-analysis of observational studies was therefore employed to examine the issues further. It was hypothesized that whole grain consumption was inversely associated with MetS, whereas refined grain consumption was positively associated with MetS.

## Materials and Methods

### Search Strategy

We conducted this meta-analysis according to the Preferred Reporting Items for Systematic review and Meta-analyses (PRISMA) guidelines (32). The PubMed, Web of Science and Embase database were searched until March 2021 (without restriction for inclusion time), by a series of logic combinations of keywords related to metabolic syndrome (“metabolic syndrome”) and grain (“grain,” “grains,” “rice,” “bread,” “breads,” “wheat,” “wheats,” “rye,” “cereal,” “cereals”). No language restrictions were set in the search strategy. We first screened the titles and abstracts of all of the articles to identify eligible studies and then read the full articles to include eligible studies. Moreover, the reference lists from retrieved articles were reviewed to identify additional studies.

### Study Selection

Two researchers (YZ and JD) reviewed the titles, abstracts and full texts of all retrieved studies independently. Disagreements were resolved by discussions and mutual-consultations. The potentially eligible articles were selected through full text review in line with the inclusion and exclusion criteria according to PICOS strategy. The included studies were required to meet the following criteria: (1) the participants were general population; (2) the exposure of interest was the consumption of whole grain or refined grain; (3) the comparison was the highest vs. lowest category of exposure; (4) the outcomes included the MetS; (5) observational studies in general population. The exclusion criteria were as follows: (1) duplicated or irrelevant articles; (2) reviews, letters or case reports; (3) randomized controlled trials; (4) non-human studies.

### Data Extraction

Two researchers extracted the data (YZ and JD) independently. Disagreements were resolved by consensus. The information about first author, year of publication, location, age, gender, sample size, study design, exposure assessment, category of exposure, effect estimates for MetS, adjustments, and diagnostic criteria of MetS was collected. The corresponding effect estimates adjusted for the maximum number of confounding variables with corresponding 95% CIs for the highest vs. lowest level were extracted. For the studies that reported the effect estimates by gender, the pooled effect estimates were calculated. In addition, Huang presented the data as southern and northern China, and they were processed independently (30). Rice/white rice was also processed as refined grain (23, 25–27).

### Quality Assessment

Quality assessment was conducted according to the Newcastle-Ottawa (NOS) criteria for non-randomized studies, which is based on three broad perspectives: the selection process of study cohorts, the comparability among different cohorts, and the identification of either the exposure or outcome of study cohorts. Disagreements with respect to the methodological quality were resolved by discussion and mutual-consultation. In the current study, we considered a study awarded seven or more stars as a high-quality study (33).

### Statistical Analyses

RR was considered as the common measure of the associations of whole grain or refined grain consumption with MetS, and OR and HR was directly converted into RR. The I^2^ statistic, which measures the percentage of the total variation across studies due to heterogeneity, was also examined (I^2^ > 50% was considered heterogeneity). If significant heterogeneity was observed among studies, the random-effects model was used; otherwise, the fixed effects model was utilized. Begg's tests were performed to assess the publication bias (34), and statistical analyses were performed using STATA version 11.0 (StataCorp LP, College Station, Texas). A *p*-value < 0.05 was accepted as statistically significant. Subgroup analysis for study design, diagnostic criteria of MetS, sample size, exposure assessment, study quality and adjustment of BMI and energy, were conducted.

## Results

### Study Identification and Selection

**Figure 1:**
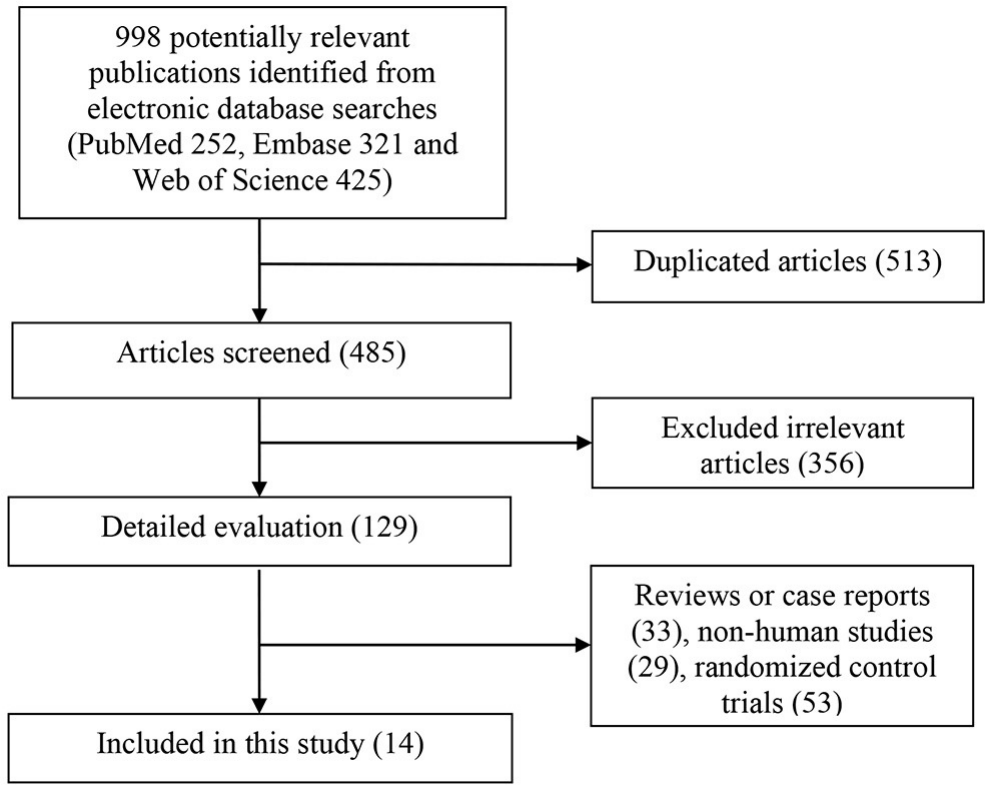
Flow chart for the identification of studies that were included in this meta-analysis.

The detailed flow diagram of study identification and selection was presented in Figure 1. A total of 998 potentially relevant articles (PubMed 252, Embase 321 and Web of Science 425) were retrieved during the initial literature search. After eliminating 513 duplicated articles, 485 articles were screened by titles and abstracts. Three hundred fifty six irrelevant studies were excluded thereafter. Then, 33 reviews, case reports or letters, 29 non-human studies, and 53 randomized control trials were removed. Eventually, a total of 14 studies were identified for this meta-analysis.

### Study Characteristics

Table 1 shows the main characteristics of the included studies. These studies were published between 2004 and 2020, which involved seven cross-sectional and seven prospective cohort studies. Ten studies were performed in the Asian countries [Korea (24, 25, 28, 31), Iran (19, 27), Japan (26), India (22) and China (23, 30)], three studies were conducted in US (18, 20, 21), and one study was conducted in Chile (29). Thirteen articles included both male and female participants (18–25, 27–31), whereas 1 study included only male participants (26). The sample size ranged from 535 to 15,972 for a total number of 61,431. Food frequency questionnaire (FFQ) was utilized in 11 studies (18, 19, 21–24, 26, 27, 29–31) and three studies employed recall record (24 h or 3 days) (20, 25, 28). Ten (18–22, 24, 25, 28, 30, 31) and four (23, 26, 27, 29) studies were defined as high and low-quality study, respectively. The diagnostic criteria for MetS were National Cholesterol Education Program-Adult Treatment Panel III (NCEP ATP III) in nine (18–20, 22, 26–29, 31), International Diabetes Foundation (IDF) in three (23, 24, 30), American Heart Association (AHA) (21, 25) in two studies, respectively.

**Table 1:** Characteristics of the individual studies included in this meta-analysis.

**First authoryear of publication**	**Location**	**Ageyears**	**Gender**	**Sample Size**	**Study design**	**Exposure assessment**	**Category of Exposure**	**Effect estimates for MetS (95%CI)**	**Adjustments**	**Diagnostic criteria of MetS**	**NOS**
Mckeown (18)	US	26–82	Both	2,834	Cross-sectional	126-item FFQ	Whole grain		Sex, age, cigarette dose, total energy intake, alcohol intake, percentage saturated fat, percentage polyunsaturated fat, multivitamin use, and physical activity	NCEP ATP III	7
							Quintile 1Quintile 2Quintile 3Quintile 4Quintile 5	1.00.81 (0.60, 1.08)1.09 (0.82, 1.44)0.82 (0.61, 1.10)0.67 (0.48, 0.91)			
							Refined grain				
							Quintile 1Quintile 2Quintile 3Quintile 4Quintile 5	1.01.13 (0.84, 1.52)1.01 (0.74, 1.38)1.03 (0.75, 1.42)0.76 (0.53, 1.09)			
Esmaillzadeh (19)	Iran	18–74	Both	827	Cross-sectional	132-item FFQ	Whole grain		Age, total energy intake, energy from fat, use of blood pressure medication, use of estrogen, smoking, physical activity, consumption of meats and fish, fruit and vegetables	NCEP ATP III	8
							Quartile 1Quartile 2Quartile 3Quartile 4	1.00.84 (0.79, 0.89)0.76 (0.69, 0.82)0.68 (0.60, 0.78)			
							Refined grain				
							Quartile 1Quartile 2Quartile 3Quartile 4	1.01.68 (1.26, 2.31)1.92 (1.48, 2.58)2.25 (1.80, 2.84)			
Sahyoun (20)	US	60–98	Both	535	Cross-sectional	3-day food record (single recall)	Whole grain		Age, sex, race, educational attainment, marital status, smoking, alcohol intake, exercise, BMI, energy intake, percentage saturated fatty acid intake and use of antihypertensive or lipid-lowering medication	NCEP ATP III	7
							Quartile 1Quartile 2Quartile 3Quartile 4	1.00.58 (0.35, 0.97)0.41 (0.24, 0.69)0.46 (0.27, 0.79)			
							Refined grain				
							Quartile 1Quartile 2Quartile 3Quartile 4	1.01.17 (0.69, 1.97)1.57 (0.91, 2.68)2.16 (1.20, 3.87)			
Lutsey (21)	US	45–64	Both	15,972	Cohort	66-item FFQ	Whole grain		Age, sex, race, education, center, and total calories, smoking status, pack-years, physical activity, meat, dairy, fruits and vegetables.	AHA	8
							Quintile 1Quintile 2Quintile 3Quintile 4Quintile 5	1.01.02 (0.92, 1.13)1.06 (0.96, 1.18)1.02 (0.92, 1.14)1.02 (0.92, 1.14)			
							Refined grain				
							Quintile 1Quintile 2Quintile 3Quintile 4Quintile 5	1.00.92 (0.83, 1.02)0.95 (0.86, 1.06)0.95 (0.85, 1.06)0.89 (0.78, 1.01)			
Radhika (22)	India	≥20	Both	2,042	Cross-sectional	222-item FFQ	Refined grain		Age, sex, smoking, alcohol, BMI, total energy, legumes, visible fats and oils, dairy products, sugars, fruits and vegetables, tubers, fish and seafoods, and nuts and oil seeds	NCEP ATP III	8
							Quartile 1Quartile 2Quartile 3Quartile 4	1.03.37 (2.13, 5.31)4.33 (2.72, 6.90)7.83 (4.72, 12.99)			
Shi (23)	China	>20	Both	1,231	Cohort	33-item FFQ	Refined grain		Smoking, drinking, active commuting, leisure time physical activity, education, occupation, energy intake	IDF	6
							<200 g201–400 g>401 g	1.00.70 (0.39, 1.26)0.76 (0.43, 1.36)			
Baik (24)	Korea	40–69	Both	5,251	Cohort	103-item FFQ	Whole grain		Age, sex, income, occupation, education, smoking status, alcohol intake, quartiles of MET-hours/day, study sites, FTO genotypes, quartiles of energy intake	IDF	7
							Quintile 1Quintile 2Quintile 3Quintile 4Quintile 5	1.0NA0.96 (0.81, 1.14)1.10 (0.84, 1.45)0.96 (0.75, 1.22)			
							Refined grain				
							Quintile 1Quintile 2Quintile 3Quintile 4Quintile 5	1.00.82 (0.68, 0.99)1.00 (0.80, 1.25)0.87 (0.68, 1.12)0.79 (0.59, 1.04)			
Son (25)	Korea	20–64	Both	5,830	Cross-sectional	24 h recall (single recall)	Refined grain		Age, energy, sex, BMI, alcohol, smoke, income, activity	AHA	7
							Quartile 1Quartile 2Quartile 3Quartile 4	1.01.02 (0.73, 1.42)1.01 (0.73, 1.40)1.09 (0.77, 1.53)			
Watanabe (26)	Japan	40–74	Male	6,095	Cohort	29-item FFQ	Refined grain <300 g/day 300–450 g/day >450 g/day	1.01.05 (0.92, 1.21)1.19 (0.79, 1.81)	Age, egg intake, vegetable intake, milk intake, sugary beverage intake, alcoholic beverage intake, family structure, daily physical activity, checking body weight <3 times per week, a gain of ≥10 kg in body weight since the age of 20, eating quickly, having dinner within 2 h of going to bed more than three times a week, snacking after dinner three or more times per week and skipping breakfast three or more times per week	NCEP ATP III	5
Bahadoran (27)	Iran	19–70	Both	2,799	Cohort	168-item FFQ	Refined grain		Age, sex, BMI, energy intake, carbohydrate, protein and fiber	NCEP ATP III	6
							Quartile 1Quartile 2Quartile 3Quartile 4	1.01.11 (0.72, 1.72)1.23 (0.79, 1.89)1.66 (1.04, 2.66)			
Song (28)	Korea	30–65	Both	6,845	Cross-sectional	24 h recall (single recall)	Whole grain		Age, living area, education, smoking status, current alcohol intake, vigorous physical activity and total energy intake.	NCEP ATP III	8
							Quintile 1Quintile 2Quintile 3Quintile 4Quintile 5	1.00.99 (0.70, 1.41)1.26 (0.88, 1.79)1.25 (0.88, 1.76)1.15 (0.82, 1.61)			
							Refined grain				
							Quintile 1Quintile 2Quintile 3Quintile 4Quintile 5	1.00.87 (0.62, 1.21)1.12 (0.80, 1.56)1.21 (0.87, 1.70)1.02 (0.75, 1.40)			
Dussaillant (29)	Chile	>18	Both	2,561	Cross-sectional	FFQ	Whole grain		Age, gender, education, physical activity, BMI	NCEP ATP III	6
							<1 serving/day≥1 serving/day	1.01.78 (1.09, 2.92)			
Huang South (30)	China	18–75	Both	1,804	Cohort	74-item FFQ	Whole grain		Gender, age, marital status, income level, urbanicity index, BMI, smoking, alcohol, physical activity, TEI, vegetable, fruit, red meat consumption	IDF	8
							Quartile 1Quartile 2Quartile 3Quartile 4	1.01.02 (0.70,1.47)1.19 (0.82,1.72)1.28 (0.87,1.90)			
							Refined grain				
							Quartile 1Quartile 2Quartile 3Quartile 4	1.01.01 (0.41, 2.49)1.05 (0.53, 2.06)1.18 (0.44, 3.16)			
HuangNorth (30)	China	18–75	Both	1,088	Cohort	74-item FFQ	Whole grain		Gender, age, marital status, income level, urbanicity index, BMI, smoking, alcohol, physical activity, TEI, vegetable, fruit, red meat consumption	IDF	8
							Quartile 1Quartile 2Quartile 3Quartile 4	1.01.06 (0.71,1.58)0.77 (0.50,1.14)0.72 (0.47,1.11)			
							Refined grain				
							Quartile 1Quartile 2Quartile 3Quartile 4	1.00.80 (0.59, 1.07)0.93 (0.69, 1.26)0.95 (0.68, 1.33)			
Kang (31)	Korea	40–69	Both	5,717	Cohort	103-item FFQ	Male		Age, education level, household income, smoking status, alcohol intake, physical activity, BMI, LDL cholesterol, total energy intake, vegetable intake, fruit intake, meat intake and dairy intake	NCEP ATP III	9
							Whole grain				
							<1 servings/day1–3 servings/day≥3 servings/day	1.00.51 (0.43, 0.61)0.51 (0.41, 0.63)			
							Refined grain				
							<1 servings/day1–3 servings/day≥3 servings/day	1.01.15 (0.98, 1.36)1.63 (1.31, 2.03)			
							Female				
							Whole grain				
							<1 servings/day1-3 servings/day≥3 servings/day	1.00.58 (0.49, 0.68)0.73 (0.60, 0.90)			
							Refined grain				
							<1 servings/day1-3 servings/day≥3 servings/day	1.00.96 (0.82, 1.12)2.25 (1.82, 2.78)			

### Association Between Whole Grain Consumption and MetS

**Figure 2:**
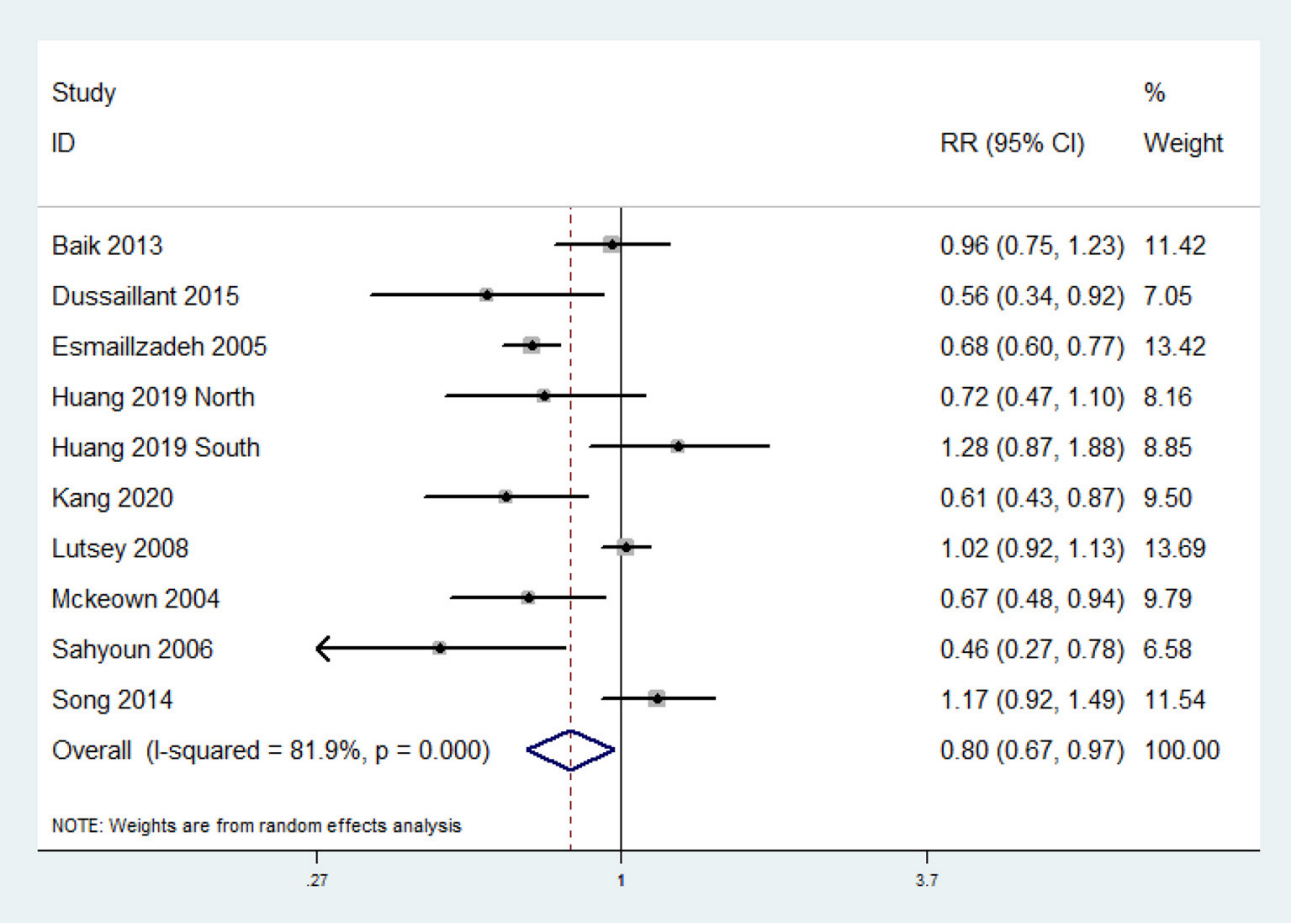
Forest plot of meta-analysis: Overall multi-variable adjusted RR of MetS for the highest vs. the lowest category of whole grain consumption.

The overall multi-variable adjusted RR evidenced an inverse association between whole grain consumption and MetS (RR = 0.80, 95%CI: 0.67–0.97; *P* = 0.02) (Figure 2). A substantial level of heterogeneity was observed among studies (*P* < 0.001, *I*^2^ = 81.9%). No evidence of publication bias was observed among the included studies according to the Begg rank-correlation test (*P* = 0.152). In addition, such findings were obtained only in cross-sectional (RR = 0.71, 95%CI: 0.53–0.95; *P* = 0.02), NCEP ATP III (RR = 0.69, 95%CI: 0.54–0.89; *P* = 0.004), FFQ (RR = 0.80, 95%CI: 0.65–0.97; *P* = 0.03), adjustment of BMI (RR = 0.69, 95%CI: 0.49–0.98; *P* = 0.04) and energy intake (RR = 0.75, 95%CI: 0.59–0.96; *P* = 0.01) studies (Table 2).

**Table 2:** Subgroup analyses of whole grain consumption and MetS.

**Stratification**	**Number of studies**	**Pooled RR**	**95% CI**	***P-*value**	**Heterogeneity**
All	9	0.80	0.67, 0.97	*P* = 0.02	*P* < 0.001; *I*^2^ = 82%
**Study design**					
Cross-sectional	5	0.71	0.53, 0.95	*P* = 0.02	*P* < 0.001; *I*^2^ = 80%
Cohort	4	0.91	0.74, 1.12	*P* = 0.37	*P* = 0.02; *I*^2^ = 65%
**Diagnostic criteria of MetS**					
NCEP ATP III	6	0.69	0.54, 0.89	*P* = 0.004	*P* = 0.001; *I*^2^ = 76%
Other	3	1.01	0.92, 1.10	*P* = 0.85	*P* = 0.25; *I*^2^ = 26%
**Exposure assessment**					
FFQ	7	0.80	0.65, 0.97	*P* = 0.03	*P* < 0.001; *I*^2^ = 82%
Other	2	0.76	0.30, 1.89	*P* = 0.55	*P* = 0.002; *I*^2^ = 90%
**Study quality**					
High-quality	8	0.83	0.68, 1.00	*P* = 0.05	*P* < 0.001; *I*^2^ = 83%
Low-quality	1	0.56	0.34, 0.92	/	/
**Adjustment of BMI**					
Adjusted	4	0.69	0.49, 0.98	*P* = 0.04	*P* = 0.01; *I*^2^ = 69%
Unadjusted	5	0.88	0.70, 1.11	*P* = 0.29	*P* < 0.001; *I*^2^ = 88%
**Adjustment of energy intake**					
Adjusted	6	0.75	0.59, 0.96	*P* = 0.01	*P* < 0.001; *I*^2^ = 79%
Unadjusted	3	0.90	0.67, 1.20	*P* = 0.45	*P* = 0.03; *I*^2^ = 67%

### Association Between Refined Grain Consumption and MetS

**Figure 3:**
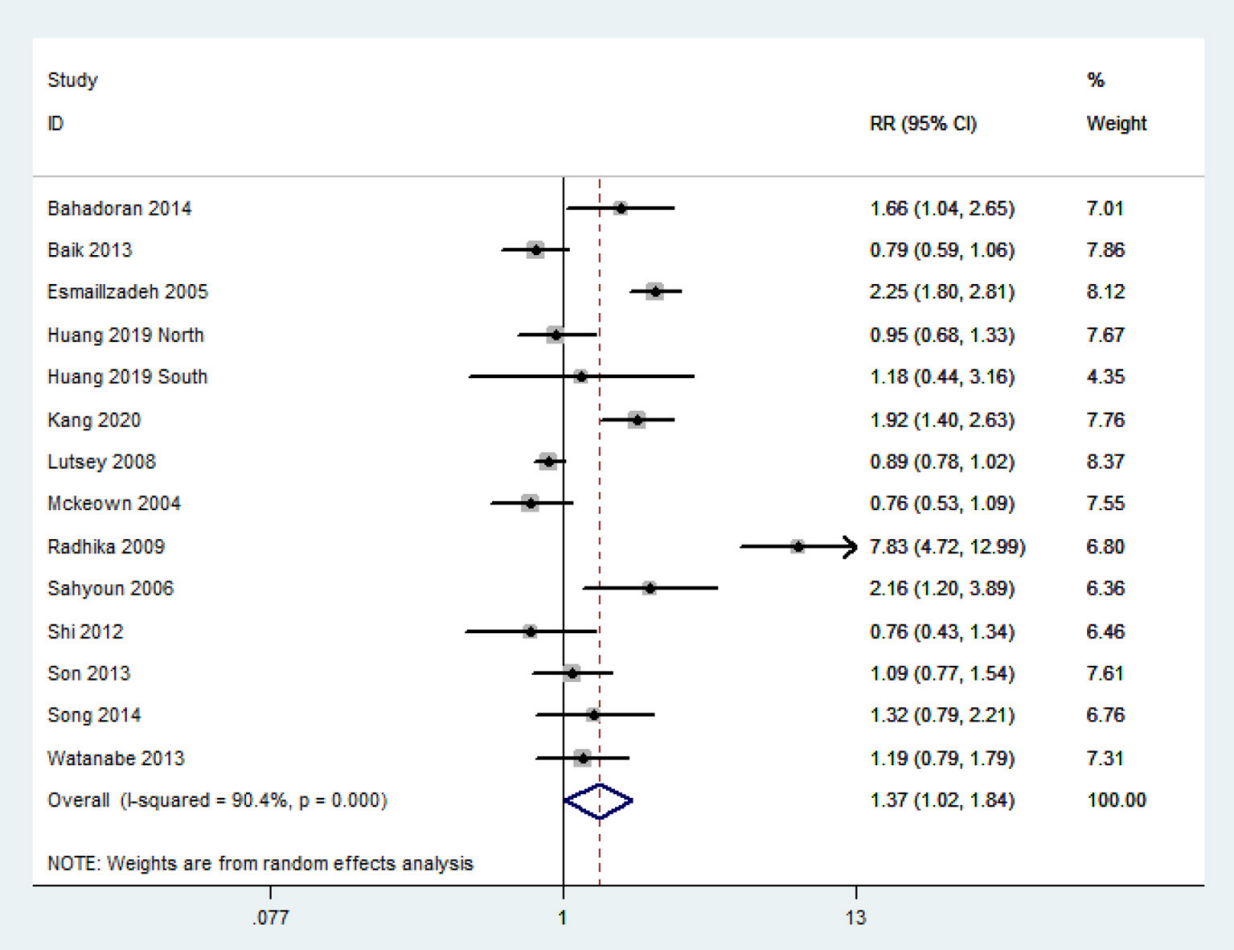
Forest plot of meta-analysis: Overall multi-variable adjusted RR of MetS for the highest vs. the lowest category of refined grain consumption.

The overall multi-variable adjusted RR demonstrated that refined grain consumption was positively associated with MetS (RR = 1.37, 95%CI: 1.02–1.84 *P* = 0.036) (Figure 3). A substantial level of heterogeneity was observed among studies (*P* < 0.001, *I*^2^ = 90.4%). No evidence of publication bias was observed among the included studies according to the Begg rank-correlation test (*P* = 0.324). In addition, such findings were obtained only in cross-sectional (RR = 1.84, 95%CI: 1.03–3.28; *P* = 0.04), NCEP ATP III (RR = 1.84, 95%CI: 1.22–2.79; *P* = 0.004), high-quality studies (RR = 1.44 95%CI: 1.01–2.04; *P* = 0.04), adjustment of BMI (RR = 1.82, 95%CI: 1.10–3.02; *P* = 0.02) and energy intake (RR = 1.54, 95%CI: 1.04–2.28; *P* = 0.02) studies (Table 3).

**Table 3:** Subgroup analyses of refined grain consumption and MetS.

**Stratification**	**Number of studies**	**Pooled RR**	**95% CI**	***P-*value**	**Heterogeneity**
All studies	13	1.37	1.02, 1.84	*P* = 0.04	*P* < 0.001; *I*^2^ = 90%
**Study design**					
Cross-sectional	6	1.84	1.03, 3.28	*P* = 0.04	*P* < 0.001; *I*^2^ = 93%
Cohort	7	1.10	0.86, 1.40	*P* = 0.46	*P* < 0.001; *I*^2^ = 75%
**Diagnostic criteria of MetS**					
NCEP ATP III	8	1.84	1.22, 2.79	*P* = 0.004	*P* < 0.001; *I*^2^ = 89%
Other	5	1.02	1.00, 1.04	*P* = 0.12	*P* = 0.22; *I*^2^ = 27%
**Exposure assessment**					
FFQ	10	1.36	0.95, 1.93	*P* = 0.09	*P* < 0.001; *I*^2^ = 92%
Other	3	1.31	1.01, 1.69	*P* = 0.04	*P* = 0.15; *I*^2^ = 48%
**Study quality**					
High-quality	10	1.44	1.01, 2.04	*P* = 0.04	*P* < 0.001; *I*^2^ = 92%
Low-quality	3	1.18	0.78, 1.76	*P* = 0.43	*P* = 0.12; *I*^2^ = 54%
**Adjustment of BMI**					
Adjusted	6	1.82	1.10, 3.02	*P* = 0.02	*P* < 0.001; *I*^2^ = 89%
Unadjusted	7	1.06	0.75, 1.51	*P* = 0.74	*P* < 0.001; *I*^2^ = 90%
**Adjustment of energy intake**					
Adjusted	10	1.54	1.04, 2.28	*P* = 0.03	*P* < 0.001; *I*^2^ = 91%
Unadjusted	3	0.92	0.82, 1.04	*P* = 0.17	*P* = 0.56; *I*^2^ = 0%

## Discussions

In this study, a total of 14 observational studies were identified. Our pooled analysis showed that whole grain consumption was negatively associated with MetS, whereas refined grain consumption was positively associated with MetS.

The opposite results with regard to the whole grain and refined grain consumption could be explained by several biological mechanisms. First, the glycemic index (GI) and the glycemic load (GL), which are both determined by the amount of carbohydrates consumed, contribute to the glycemic response directly (35). It was reported that GIs and GLs were associated with a higher risk of MetS, which was independent of diabetes mellitus (36). Compared with refined grain, whole grain tends to be absorbed slowly with a relatively low GI. On the contrary, refined grain is abundant in carbohydrate content, which leads to a higher dietary GL (37). Second, whole grain is rich in dietary fiber, trace minerals, and phytochemicals (37). These nutrients and food components were considered to be beneficial for MetS (38, 39). However, the nutrient components of refined grain were lost during the refining process (22).

Our results were supported by several randomized control trials directly. Jackson et al. (14) showed that the replacing refined grain by whole grain in weight-loss diet could reduce glucose directly. Moreover, Giacco et al. (15) further indicated that a 12-weeks of whole grain intervention could reduce postprandial insulin and triglycerides responses in MetS (also compared to refined grain) (15). On the other hand, some fundamental experimental study should also be emphasized. Both Hao et al. (16) and Yen et al. (17) demonstrated that pre-germinated brown rice extract could ameliorate high-fat diet-induced MetS model. Above all, the results of our study were partly supported by the corresponding clinical and experimental evidence.

Generally speaking, whole grain referred to barley, multigrain and ground mixed grain, whereas refined grain included white rice, noodles and bread. Interestingly, several studies have considered the grain consumption as a whole (without subtype specification). Unsurprisingly, no significant relationship was obtained in these studies (40–43). It could be attributed to the synergistic effect of whole grain and refined grain consumption on MetS. Of note, our result was only confirmed in cross-sectional studies both for whole grain and refined grain. Although the reliability of our results may be influenced by the substantial level of heterogeneity, the potential different effect of grain consumption on the prevalence or risk of MetS could not be ignored, it was still an open question for MetS prevention. Moreover, the inconsistent result with regard to diagnostic criteria of MetS, exposure assessment and study quality (for refined grain) was also acquired. FFQ seems to be more reliable and precise for dietary assessment, and the NCEP ATP III criteria may be suitable for considering the effect of grain consumption. In addition, the quality of study may also influence the results. High consumption of whole-grain foods is associated with lower BMI in a dose-dependent manner (44), and BMI is considered as an important factor in MetS (1). Moreover, grain consumption is closely associated with appetite and energy intake (45), and a low reported energy intake is also reported to be associated with MetS (46). Indeed, the inconsistent result with regard to the adjustment of BMI and energy intake was obtained in our study. Therefore, further studies are required to consider BMI and energy intake as confounding factors. It should also be noted that the heterogeneity of our study cannot be ignored, especially for the exposure assessment. A semi-quantitative FFQ was utilized in most studies (18, 19, 21–24, 26, 27, 29–31), whereas several studies used recall record (20, 25, 28). On the other hand, the definition of whole grain or refined grain may vary greatly among individuals. For example, refined grain always refers to rice, noodles or bread etc, but several studies only considered rice/white rice (23, 25–27). These inconsistent exposure assessments could cause significant heterogeneity and inaccuracies in the interpretation of the results. Of note, the plasma level of alkylresorcinols, which was served as a reliable marker of whole grain consumption (14), was unfortunately ignored in all the included studies. Therefore, more well-designed prospective cohort studies are still needed.

Our study has several strengths. First, this is the first meta-analysis of observational study aiming at the associations of whole grain and refined grain consumption with MetS. Second, the included studies were analyzed based on adjusted results and large samples. Third, our result might be helpful to better consider the diet effect on MetS. We should also acknowledge the limitations of the present study. First, the substantial level of heterogeneity might have distorted the results. Second, due to the limitation of relevant literature, only a limited number of observational studies were identified for this meta-analysis. Third, the classification of exposure may vary greatly among individuals. Fourth, the diagnostic criteria of MetS and the selection of adjusted factors were not uniform. Last but not the least, a subgroup for gender could not be performed since very limited study specified the effect estimates by gender. These limitations might weaken the significance of this study.

## Conclusions

The existing evidence suggests that whole grain consumption is negatively associated with MetS, whereas refined grain consumption is positively associated with MetS. More well-designed prospective cohort studies are needed to elaborate the concerned issues further.

## Data Availability Statement

The raw data supporting the conclusions of this article will be made available by the authors, without undue reservation.

## Author Contributions

YZ conceived the idea and performed the statistical analysis. YZ, HG, and JD drafted this meta-analysis. HG and JD selected retrieved relevant papers. HG and JL assessed each study. YZ was the guarantor of the overall content. All authors revised and approved the final manuscript.

## Conflict of Interest

The authors declare that the research was conducted in the absence of any commercial or financial relationships that could be construed as a potential conflict of interest.
